# Micronutrient therapy for pyrroluria: a retrospective analysis of patient acceptance

**DOI:** 10.1007/s00404-025-08252-8

**Published:** 2026-01-06

**Authors:** Lena Zingg, Elena Pavicic, Norman Bitterlich, Petra Stute

**Affiliations:** 1 Faculty of Medicine, University of Bern, Bern, Switzerland; 2 Department of Obstetrics and Gynecology, University Hospital of Bern, Bern, Schweiz; 3 Graduate School for Health Sciences, University of Bern, Bern, Schweiz; 4 Freelance Statistician “Statistics – analysis, Consulting, Training”, Chemnitz, Germany

**Keywords:** Micronutrients, Pyrroluria, Kryptopyrroles, Therapy Acceptance, Women’s Health

## Abstract

**Purpose:**

Pyrroluria is a proposed metabolic condition that remains controversial and insufficiently supported by empirical evidence within conventional medical practice and research. The present study evaluates patient acceptance of micronutrient therapy prescribed for pyrroluria in a clinical setting. The aim is not to validate the condition but to document patient experiences with a therapy commonly used in complementary practice.

**Methods:**

This retrospective cohort study was conducted in the Department of Gynecology at the University Hospital of Bern in 2022/2023. The study included patients who tested positive for pyrroluria and received micronutrient therapy. The primary outcome was the overall acceptance of therapy, assessed using the validated ACCEPT© questionnaire.

**Results:**

The mean score for overall therapy acceptance was 74 ± 34 out of a maximum of 100 points, indicating a high level of acceptance. Micronutrient therapy was also well accepted in terms of side effects (85 ± 27), treatment constraints (71 ± 24), long-term use (85 ± 20), and therapy regimen (84 ± 21). The perceived efficacy of the therapy received a score of 63 ± 34, which did not reach statistical significance (p = 0.7).

**Conclusions:**

Micronutrient therapy was well tolerated and accepted by patients with pyrroluria, underscoring its potential as a low-risk adjunctive intervention. However, the perceived treatment efficacy was modest, and no conclusions about the biological validity of pyrroluria or the effectiveness of the therapy can be drawn. Rigorous, placebo-controlled trials are needed to evaluate the therapeutic value of micronutrients in this context. Furthermore, substantial scientific investigation is required to determine whether pyrroluria constitutes a valid clinical entity within conventional medicine.

**Supplementary Information:**

The online version contains supplementary material available at 10.1007/s00404-025-08252-8.

## Introduction

### What this study adds to the clinical work

**Table:** 

Pyrroluria is a proposed metabolic condition largely unrecognized in conventional medicine, yet it is widely discussed in complementary and integrative medicine due to its possible association with a broad range of symptoms.
Micronutrient supplementation is commonly used in this context, and understanding ist acceptance may help improve patient management and therapy, particularly in complex multisystemic disorders.

Pyrroluria is a hypothesized metabolic condition [1–3] first described in the 1960s by D. G. Irvine in association with psychiatric disorders [4]. Despite early interest, the concept has not been substantiated by robust biochemical or epidemiological evidence [5], and it has received limited recognition within mainstream medical research [1, 2]. Consequently, to date, pyrroluria remains a controversial and insufficiently validated diagnosis.

The condition is characterized by increased urinary excretion of kryptopyrroles (2,4-dimethyl-3-ethylpyrrole) [6–10], a group of by-products thought to arise from disturbances in heme metabolism [11, 12]. It is therefore often classified within the broader group of porphyrioses [8, 11–13].

Reported symptoms attributed to pyrroluria are broad and nonspecific [1, 2], including psychiatric and neurological features such as schizophrenia, depression, mood instability, anxiety and attentional difficulties [4, 6, 7, 9, 10, 12, 14–18], as well as somatic manifestations like chronic fatigue, endocrine dysfunction, immune irregularities and impaired stress tolerance [1–3, 19].

A prevailing hypothesis proposes, on the one hand, that excess pyrroles themselves exert a deleterious effect on the CNS [10, 15, 16, 20]. Disturbances in acetylcholinergic pathways have also been described, which could explain both CNS impairment and altered muscle function [20]. On the other hand, excessive pyrroles are thought to bind essential micronutrients [21]—above all vitamin B6, zinc and manganese [1, 2, 21]—leading to their accelerated depletion with increased urinary excretion [21]. Such losses can result in micronutrient deficiencies [21] and thereby disrupt neurotransmitter synthesis, mitochondrial energy metabolism and detoxification pathways [22–27], providing a mechanistic explanation for the broad overlap of psychiatric, metabolic and somatic symptoms [26, 27]. This could also explain why targeted micronutrient supplementation has been reported to alleviate symptoms in affected patients [22–25, 28].

Although these findings were not widely taken up in academic medicine [1, 2], they continue to inform current clinical practice in complementary and integrative settings, as well as among patient-led initiatives [1–3, 28–32]. Prevalence estimates as high as 10% in certain populations have been reported, with up to 90% of cases occurring in females [1, 2, 6]. If accurate, these estimates underscore the potential clinical relevance of pyrroluria and suggest that a substantial proportion of patients with psychiatric or somatic disorders may be affected by an overlooked metabolic condition [1–3, 24–29].

While micronutrient supplementation is recommended [1, 2, 22, 24–28, 30–33], systematic evaluations of its acceptance, efficacy and long-term outcomes remain scarce [34]. The present study aims to address this gap by examining the acceptance of micronutrient supplementation among individuals diagnosed with pyrroluria. By capturing patient-reported outcomes, this work may help inform future research priorities, including the critical need for well-designed studies to evaluate both the biological basis of pyrroluria and the utility of associated therapeutic approaches. It may help stimulate renewed scientific interest in pyrroluria and its possible role as an under-recognized but potentially modifiable factor in complex, multisystemic disorders. If current hypotheses and prevalence estimates prove correct, a relatively simple, safe and accessible therapeutic strategy could hold substantial benefits for a large number of affected individuals.

## Material and methods

### Study design

This was a single-center, retrospective cohort study conducted at the Department of Gynecology and Obstetrics, Inselspital, University Hospital of Bern, Switzerland in 2022/2023. The study population consisted of patients with a confirmed diagnosis of pyrroluria (diagnostic criteria below) who had received micronutrient therapy. Micronutrient therapy was administered in the form of standardized capsules. Each capsule contained the following ingredients:

- Calcium pantothenate: 109 mg (100 mg pure pantothenic acid)
- Zinc gluconate: 70 mg (10 mg elemental zinc)
- Pyridoxal-5-phosphate: 50 mg
- Coenzyme Q10: 30 mg
- Chromium picolinate: 0.3 mg (40 µg elemental chromium)
- Cellulose as filler and capsule shell material.

The prescribed dosage was one capsule per day.

### Diagnostics of pyrroluria

Testing was performed using a specific test [34] designed to measure urinary kryptopyrroles (zinc–P5P complex of 2,4-dimethyl-3-ethylpyrrole) in the first morning urine. Kryptopyrrole levels were normalized to creatinine, and concentration ≥ 5 mg/g creatinine were considered pathological and used as the diagnostic threshold for pyrroluria. The analysis employed a proprietary colorimetric method. After light-protected transport, urine samples undergo internal stabilization procedures due to the high light and oxidation sensitivity of pyrrole compounds. Quantification is based on a spectrophotometric measurement following a pyrrole-reactive color reaction, and results are reported as mg/g creatinine. However, the test employed is not analytically validated, and no standardized reference ranges, reproducibility data, or false-positive/false-negative rates have been established.

### Participants and recruitment

The study population consisted of patients diagnosed with pyrroluria who received micronutrient therapy. All the patients were diagnosed and treated at the Department of Gynecology and Obstetrics, Inselspital, Bern. All included patients were female and above the age of 18 years. A total of 122 patients were initially identified and received an invitation letter explaining the content, objectives and procedure of the study. In addition, the informed consent form was sent along, which was completed and returned by the patients interested in participation. 34 patients gave their informed consent to participate in the study, resulting in a response rate of 27.8%. Following the signed informed consent, the participants received the link to the online questionnaire via email. Inclusion criteria required that participants had a confirmed diagnosis of pyrroluria and were undergoing or had undergone micronutrient therapy. Excluded were people not willing or unable to complete the online questionnaire. Missing data within the ACCEPT© questionnaire were handled as follows: participants with more than 20% missing items or with missing data in any of the ACCEPT© subscale were excluded from the analysis. No item-level imputation was performed. After obtaining informed consent, nine respondents were excluded based on these criteria, leaving 25 complete datasets for the final analysis, corresponding to a response rate of 20.5%.

### Study population

Of the 122 patients with confirmed pyrroluria who were invited to participate, 34 (27.8%) provided informed consent. After exclusion of nine incomplete questionnaires, 25 complete datasets were available for final analysis. All participants were female and ≥ 18 years old and had undergone or were undergoing micronutrient therapy. Demographic characteristics of the study population are summarized in Table 1.

**Table 1:** Demographic characteristics of the study population (n = 25)

Characteristic	Category	n (%)
Education	Vocational apprenticeship	9 (36)
	High-school diploma	4 (16)
	University degree	10 (40)
	Missing data	2 (8)
Employment status	Employed (part- or full-time)	21 (84)
	Unemployed/unable to work	2 (8)
	Missing data	2 (8)
Monthly income (CHF)	< 5,000	13 (52)
	5,000–10,000	6 (24)
	> 10,000	3 (12)
	No income	1 (4)
	Missing data	2 (8)

### Data collection

Data was collected using an online questionnaire, which included the validated ACCEPT© questionnaire (Supplementary File 1) along with additional questions on the demographics of the participants (Supplementary File 2). The ACCEPT© questionnaire is a well-established tool designed to assess the acceptance of drug therapies, consisting of 25 items that evaluate how patients balance the pros and cons of their medication.

### Study instrument

The ACCEPT© questionnaire (Supplementary File 1) measures various dimensions of therapy acceptance, including general acceptance, acceptance of side effects, acceptance of restrictions, acceptance of long-term therapy, and acceptance of efficacy. General acceptance is quantified using three items, with scores presented on a scale from 0 to 100, where a higher score indicates greater acceptance. A score above 50 is considered indicative of acceptance of the therapy.

### Hypothesis and endpoints

The null hypothesis of this study posits that micronutrient supplementation as a therapy for pyrroluria is not accepted by the affected patients. The primary endpoint is general acceptance as measured by the ACCEPT© questionnaire, which is assessed using three questions. Participants were asked whether they believed the therapy had more advantages than disadvantages, whether they considered the therapy to be an acceptable solution, and whether it was worth continuing the therapy over a longer period.

The secondary endpoints of the study included the long-term acceptance of the therapy, acceptance in relation to side effects, restrictions caused by the therapy, medication intake, and perceived effectiveness of the therapy. These aspects were assessed using five additional items from the ACCEPT© questionnaire, with each endpoint comprising 3–5 specific items.

### Statistical analysis

All collected data were evaluated descriptively. A 95% confidence interval was calculated for all relevant parameters. Statistical tests were conducted using non-parametric methods due to the nature of the data distribution. Because ACCEPT© scores are bounded, non-parametric, and displayed non-normal distributions, a one-sample Wilcoxon signed-rank test was applied to compare observed scores against the theoretical midpoint (50). The threshold of 50 is defined in the scoring manual as the cut-off representing neutral acceptance; values above this midpoint indicate increasingly positive appraisal. Thus, the test evaluates whether the median acceptance score in our cohort exceeded the scale-defined neutrality point. The assumptions for the Wilcoxon test—paired differences that are symmetrically distributed around the median—were met based on exploratory distributional checks. The five ACCEPT© subdomains were analyzed as exploratory secondary outcomes. Since these analyses were not powered for confirmatory inference and aimed primarily at characterizing patterns of acceptance, no correction for multiple testing was applied.

### Ethics

This study was conducted in accordance with ethical standards and received approval from the Cantonal Ethics Committee of Bern, Switzerland (Project-ID: 2021-01026) (Supplementary File 3). All participants provided informed consent, ensuring their voluntary and informed participation in the research.

## Results

A total of 25 complete datasets were available for analysis after exclusion of incomplete questionnaires.

### Primary endpoint

The mean score for *general acceptance of therapy* was 74.2 out of 100 (95% CI: 60.1–88.3). According to the ACCEPT© questionnaire criteria (Supplementary File 1), a score above 50 points indicates acceptance of the therapy. Therefore, this result demonstrates an overall positive evaluation of the therapy. Statistical testing with the Wilcoxon signed-rank test confirmed that this difference was significant (z = –2.82, p = 0.005), thereby rejecting the null hypothesis that micronutrient therapy would not be accepted and demonstrating high acceptance levels among participants (Table 2).

**Table 2:** Primary endpoint—general acceptance of therapy according to the ACCEPT © questionnaire

Parameter	Value
Mean score	74.22
95% CI	60.12–88.32
Standard deviation	34.17
Standard error of mean	6.83
z-value (Wilcoxon test)	− 2.82
p-value (two-sided)	0.005

### Secondary endpoints

**Fig. 1:**
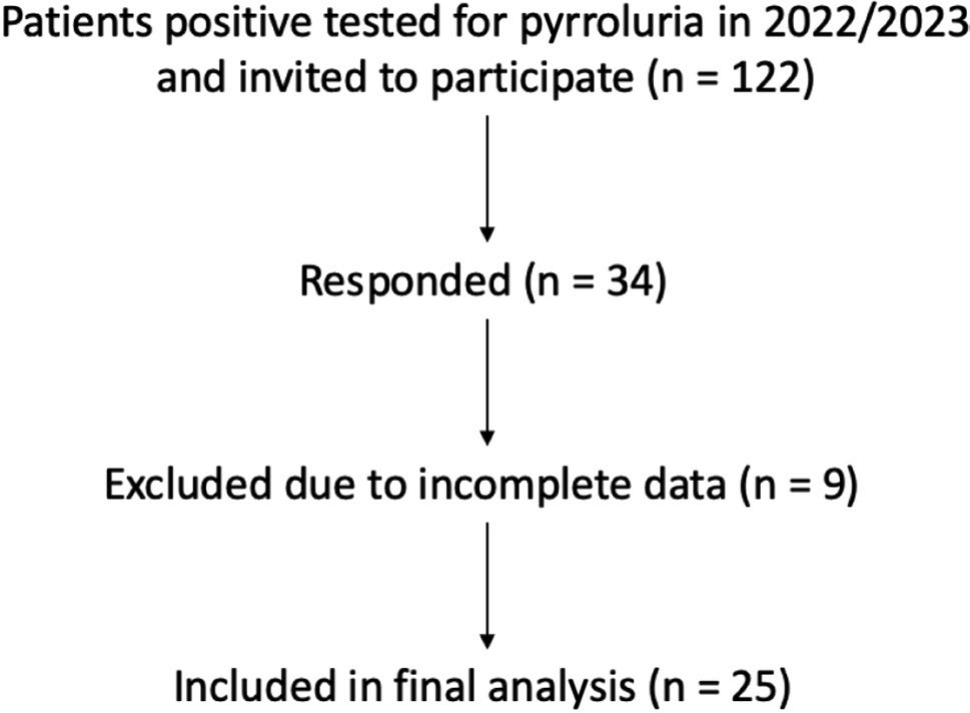
Study flow diagram

Acceptance was also assessed across five additional dimensions of the ACCEPT© questionnaire (Supplementary File 1). Regarding the secondary endpoints, statistical analysis revealed that four out of five showed statistically significant results (p < 0.05; Table 3, Fig. 1).

**Table 3:** Secondary endpoints—acceptance dimensions according to the ACCEPT © questionnaire

Dimension	Mean ± SD	p-value
Side effects	85 ± 27	< 0.001
Treatment constraints	71 ± 24	< 0.001
Long-term use	85 ± 20	< 0.001
Therapy regimen	84 ± 21	0.001
Perceived efficacy	63 ± 34	0.7

*Side effects*: Participants reported very high acceptance regarding side effects (mean 85 ± 27). This was statistically significant (p < 0.001), indicating that the majority of patients did not experience relevant or burdensome adverse reactions.

*Treatment constraints*: The acceptance of therapy-related constraints (such as remembering intake or obtaining supplements) was somewhat lower but still positive (mean 71 ± 24, p < 0.001).

*Long-term use*: The long-term feasibility of micronutrient therapy was rated highly (mean 85 ± 20, p < 0.001), suggesting that patients considered the treatment sustainable over time.

*Therapy regimen*: The practical aspects of therapy administration (e.g. form of intake, integration into daily life) also received a high score (mean 84 ± 21, p = 0.001).

**Fig. 2:**
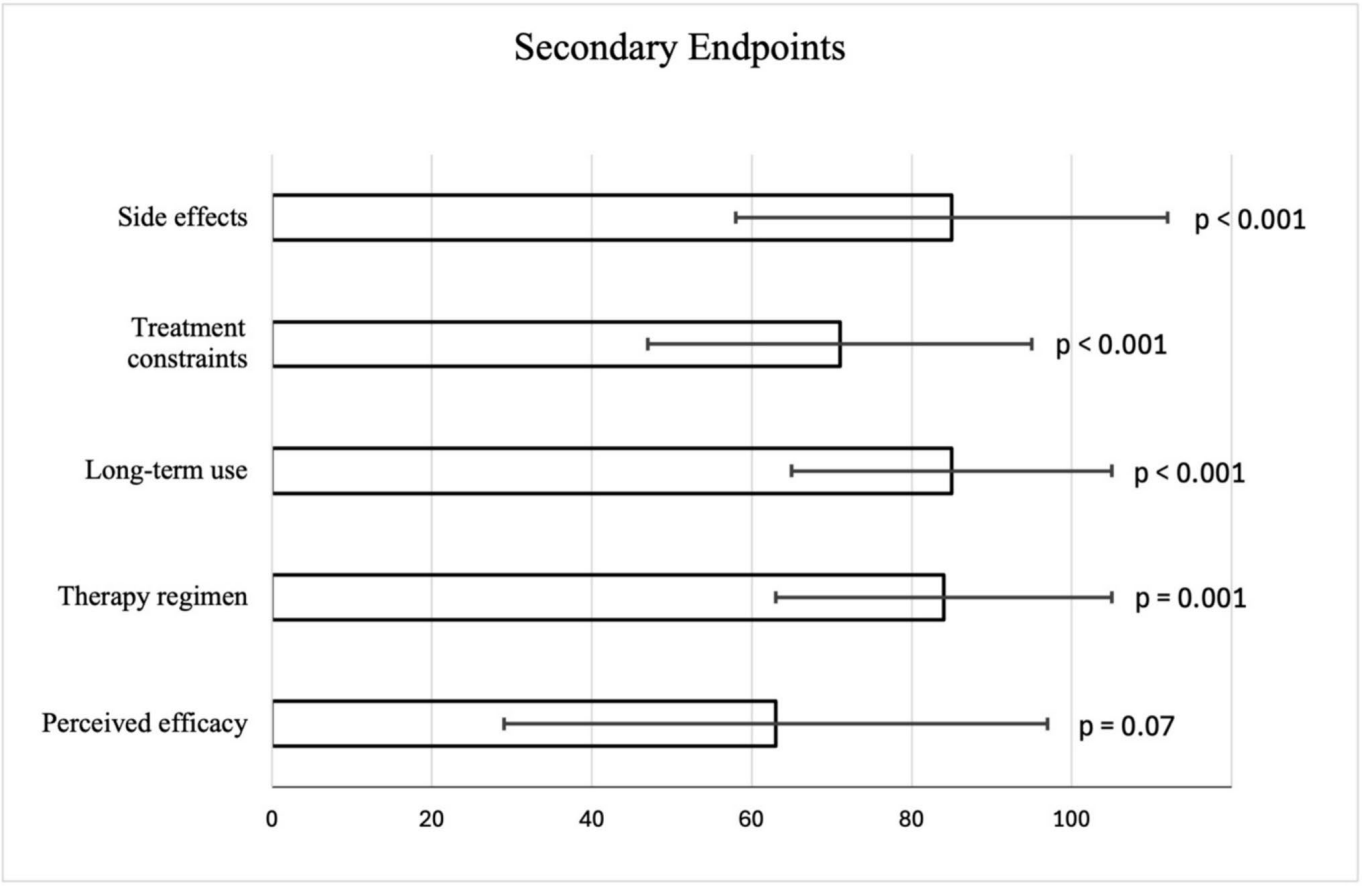
Secondary endpoints with their confidential intervals and p-value: Scores > 50 indicate acceptance; error bars show 95% confidence intervals

*Perceived efficacy*: In contrast, the dimension of perceived therapeutic efficacy achieved only a mean score of 63 ± 34 and did not reach statistical significance (p = 0.7). This finding reflects uncertainty among patients about whether the therapy had a tangible effect on their symptoms (Fig. 2).

## Discussion

Our study found a statistically significant positive acceptance of micronutrient treatment among patients diagnosed with pyrroluria, indicating that the therapeutic interventions were generally well received. This positive evaluation also extended to the secondary endpoints, which examined side effects, medication constraints, and long-term adherence. Patients reported good tolerability, manageable dosing requirements, and sustained compliance, all of which are crucial for the success of long-term metabolic interventions. The only domain without statistically significant endorsement was perceived efficacy, suggesting that while patients accept micronutrient therapy as manageable and safe, they remain uncertain about its effectiveness.

The finding that patients rated tolerance and feasibility positively could be clinically relevant. Many of the conditions associated with pyrroluria—including psychiatric disorders such as psychosis, depression, anxiety and ADHD [4, 6, 7, 9, 14, 17–19]—are often treated with pharmacological regimens that may entail substantial side effects or adherence challenges. In this context, micronutrient supplementation represents a low-risk intervention with high patient acceptability. The absence of significant adverse effects is particularly noteworthy, as it distinguishes micronutrient supplementation from many standard psychopharmacological or endocrinological treatments, where tolerability often determines long-term adherence [35]. From a clinical perspective, these results suggest that micronutrient therapy could be a valuable adjunct for patients with complex, multi-systemic complaints [1–3, 19].

However, the lower ratings for perceived efficacy underline an important gap between patient experience and scientific validation [5]. To date, much of the discourse on pyrroluria and its treatment has been carried out outside academic medicine [1–3], with a reliance on case reports, clinical anecdotes, and patient advocacy rather than on controlled trials [5]. The scientific work in the 1960s and 1970s [4, 6–16, 20–23, 25] laid important groundwork, but systematic replication and rigorous study designs remain lacking [5]. The publication *“Pyrroluria: Fact or Fiction?”* [5] provides a useful overview of the current evidence landscape and highlights that several aspects of pyrroluria—such as its biochemical basis, diagnostic reproducibility, and symptom specificity—remain insufficiently clarified [5]. These factors are indispensable for any integration of pyrroluria into convetional diagnostic frameworks. While the paper does not dismiss pyrroluria entirely, it emphasizes that the available evidence is limited [5]. Our findings align with this perspective in that we report high patient acceptance but cannot draw conclusions regarding the biological validity of the diagnosis or the efficacy of micronutrient supplementation.

Although the sample size was small, the available demographic data (Table 1) offer some insight into the characteristics of individuals seeking evaluation or support for symptoms potentially associated with pyrroluria. Participants represented a relatively well-educated group, with most having completed either a university degree or a vocational apprenticeship, and the majority were employed at least part-time. The recruitment setting (a gynecological clinic) resulted in the inclusion of only female participants. This aligns with the commonly reported, though insufficiently substantiated, higher prevalence of pyrroluria in women [1, 2]. However, our findings can therefore not be generalized to male patients and further research is needed to evaluate potential differences in presentation and prevalenc between the sexes. Over half reported a monthly income below CHF 5,000, suggesting that financial constraints may play a role in healthcare access or treatment choices for some individuals. While these findings should be interpreted cautiously due to the limited number of respondents and some missing data, they nonetheless provide useful context for understanding the population engaging with complementary or integrative approaches related to pyrroluria.

Several limitations must be acknowledged. First, the retrospective cohort design and small sample size restrict the generalizability of the findings. The response rate of 27.8% (20.5% complete cases) introduces a high risk of selection bias, as patients with higher treatment acceptance or stronger belief in the therapy may have been more likely to participate. Second, the study relied solely on patient-reported outcomes and did not include validated symptom scales, biochemical assessments, or comparator groups, limiting the ability to draw conclusions about clinical effectiveness. Third, the analysis of the five ACCEPT© subdomains as secondary outcomes introduces the possibility of type I error inflation due to multiple testing. Because these analyses were exploratory, p-values were not corrected for multiplicity and should be interpreted with caution. Fourth, a wast majority of the cited literature is outdated and/or does not match current research standards. Finally diagnostic criteria and testing procedures are not standardized, and the condition’s biochemical basis and clinical relevance remain unclear.

## Conclusion

In conclusion, micronutrient supplementation for pyrroluria is well accepted by patients and perceived as tolerable and feasible. However, perceptions of efficacy were modest, and the current study does not provide evidence for the effectiveness of the therapy or for the validity of pyrroluria as a clinical entity. These results support the necessity of further foundational research. Future work must first address whether pyrroluria represents a distinct and scientifically verifiable condition. Secondly, prospective, placebo-controlled trials are needed to further investigate effectiveness of micronutrient therapy and developing standardized and effective treatment protocols. Strengthening the evidence base in this manner is necessary before pyrroluria can be considered for broader clinical recognition or integrated into conventional medical practice.

## Supplementary Information

Below is the link to the electronic supplementary material.

Supplementary file 1 (PDF 96 KB)

Supplementary file 2 (PDF 174 KB)

Supplementary file 3 (PDF 1653 KB)

## Data Availability

The datasets generated and/or analysed during the current study are not publicly available due to patient confidentiality and institutional data protection regulations, but are available from the corresponding author on reasonable request.
