# Supplementary material for: Micronutrient therapy for pyrroluria: a retrospective analysis of patient acceptance
Source: Arch Gynecol Obstet. 2026 Jan 6;313(1):14. doi: 10.1007/s00404-025-08252-8 (PMC12774977; doi:10.1007/s00404-025-08252-8)
Supplement: Supplementary file 1 — (PDF 96 KB) [file 404_2025_8252_MOESM1_ESM.pdf]

SIE UND IHR MEDIKAMENT:
WIE LEICHT FÄLLT ES IHNEN, ES ZU AKZEPTIEREN?

Name des Medikaments, das vom Patienten eingenommen wird (vom Apotheker auszufüllen):

...

Bitte geben Sie vor dem Ausfüllen dieses Fragebogens das heutige Datum an:

/__/__/ /__/__/ /__/__/__/__/
Tag Monat Jahr

Sehr geehrte Patientin, sehr geehrter Patient

Sie haben einer Teilnahme an einer wissenschaftlichen Studie zugestimmt. Dieser Fragebogen soll uns helfen, mehr über Ihre Erfahrungen mit dem Medikament, das Sie einnehmen, zu erfahren. Bitte geben Sie den Fragebogen nach dem Ausfüllen Ihrer Apothekerin/Ihrem Apotheker zurück.
Die in diesem Fragebogen enthaltenen Informationen werden anonym und streng vertraulich behandelt.

Bitte wählen Sie beim Beantworten der folgenden Fragen diejenige Antwort aus, die Ihre Situation mit Ihrem Medikament am besten beschreibt.
Wenn Sie sich mit der Antwort nicht sicher sind, wählen Sie diejenige Antwort aus, die Ihrer Situation am nächsten kommt. Es gibt keine „richtigen“ oder „falschen“ Antworten.

Bitte füllen Sie diesen Fragebogen in einer ruhigen Umgebung und nach Möglichkeit alleine aus.

Wir danken Ihnen für Ihre Teilnahme.

Fragebogen zur Akzeptanz einer chronischen Behandlung: ACCEPT©, MAPI 2008 - Alle Rechte vorbehalten

ACCEPT - Switzerland/German - Version of 09 Oct 2018 - Mapi.
ID0295-TR-70971 / ACCEPT_AU3.0_deu-CH.doc

Bitte beantworten Sie die folgenden Fragen, indem Sie das Kästchen ankreuzen (×), das Ihre Situation mit Ihrem Medikament am besten beschreibt.

Ihr Medikament

1. Finden Sie, dass die Zubereitung Ihres Medikaments umständlich ist?

Ja, und es fällt mir nicht leicht, dies zu akzeptieren.	Ja, aber es fällt mir leicht, dies zu akzeptieren.	Nein	Mein Medikament erfordert keine Zubereitung.
☐1	☐2	☐3	☐4

2. Finden Sie, dass die Medikamenteneinnahme umständlich ist?
(Beispiele für die Medikamenteneinnahme: Schlucken, Injektion mittels Spritze, Inhalation durch die Nase usw.)

Ja, und es fällt mir nicht leicht, dies zu akzeptieren.	Ja, aber es fällt mir leicht, dies zu akzeptieren.	Nein
☐1	☐2	☐3

3. Empfinden Sie die Form Ihres Medikaments als unangenehm?
(Beispiele für Medikamentenformen: Tablette, Kapsel, Pulver in Beuteln, Spritze, Tropfen, Inhalator usw.)

Ja, und es fällt mir nicht leicht, dies zu akzeptieren.	Ja, aber es fällt mir leicht, dies zu akzeptieren.	Nein
☐1	☐2	☐3

Fragebogen zur Akzeptanz einer chronischen Behandlung: ACCEPT©, MAPI 2008 - Alle Rechte vorbehalten

Dauer Ihrer Behandlung

4. Nehmen Sie Ihr Medikament bereits seit längerer Zeit ein?

Ja, und es fällt mir nicht leicht, dies zu akzeptieren.	Ja, aber es fällt mir leicht, dies zu akzeptieren.	Nein
☐1	☐2	☐3

5. Werden Sie Ihr Medikament für längere Zeit einnehmen müssen?

Ja, und es fällt mir nicht leicht, dies zu akzeptieren.	Ja, aber es fällt mir leicht, dies zu akzeptieren.	Nein
☐1	☐2	☐3

Fragebogen zur Akzeptanz einer chronischen Behandlung: ACCEPT©, MAPI 2008 - Alle Rechte vorbehalten

Einschränkungen mit Ihrem Medikament

6. Fühlen Sie sich dadurch eingeschränkt, dass Sie sich an die Einnahme Ihres Medikaments erinnern müssen?

Ja, und es fällt mir nicht leicht, dies zu akzeptieren.	Ja, aber es fällt mir leicht, dies zu akzeptieren.	Nein
☐1	☐2	☐3

7. Fühlen Sie sich dadurch eingeschränkt, dass Sie sich die Zeit nehmen müssen, Ihr Medikament aus der Apotheke zu holen?

Ja, und es fällt mir nicht leicht, dies zu akzeptieren.	Ja, aber es fällt mir leicht, dies zu akzeptieren.	Nein
☐1	☐2	☐3

8. Fühlen Sie sich dadurch eingeschränkt, dass Sie sich daran erinnern müssen, Ihr Medikament mit sich zu nehmen?

Ja, und es fällt mir nicht leicht, dies zu akzeptieren.	Ja, aber es fällt mir leicht, dies zu akzeptieren.	Nein	Ich muss mein Medikament nie mitnehmen.
☐1	☐2	☐3	☐4

9. Fühlen Sie sich dadurch eingeschränkt, dass Sie Ihr Medikament immer bei sich haben?

Ja, und es fällt mir nicht leicht, dies zu akzeptieren.	Ja, aber es fällt mir leicht, dies zu akzeptieren.	Nein	Ich muss mein Medikament nicht immer bei mir haben.
☐1	☐2	☐3	☐4

10. Muss Ihr Medikament auf Reisen unter speziellen Bedingungen aufbewahrt werden?

Ja, und es fällt mir nicht leicht, dies zu akzeptieren.	Ja, aber es fällt mir leicht, dies zu akzeptieren.	Nein
☐1	☐2	☐3

11. Finden Sie, dass Sie viele Medikamente einnehmen müssen?

Ja, und es fällt mir nicht leicht, dies zu akzeptieren.	Ja, aber es fällt mir leicht, dies zu akzeptieren.	Nein
☐1	☐2	☐3

Fragebogen zur Akzeptanz einer chronischen Behandlung: ACCEPT©, MAPI 2008 - Alle Rechte vorbehalten

12. Können Sie Ihr Medikament diskret einnehmen?

Nein, und es fällt mir nicht leicht, dies zu akzeptieren.	Nein, aber es fällt mir leicht, dies zu akzeptieren.	Ja
☐1	☐2	☐3

13. Finden Sie, dass die regelmässige Einnahme Ihres Medikaments Teil Ihres normalen Alltags geworden ist?

Nein, und es fällt mir nicht leicht, dies zu akzeptieren.	Nein, aber es fällt mir leicht, dies zu akzeptieren.	Ja	Ich muss mein Medikament nicht regelmässig nehmen.
☐1	☐2	☐3	☐4

14. Fühlen Sie sich dadurch eingeschränkt, wie oft Sie Ihr Medikament nehmen müssen?

Ja, und es fällt mir nicht leicht, dies zu akzeptieren.	Ja, aber es fällt mir leicht, dies zu akzeptieren.	Nein
☐1	☐2	☐3

Fragebogen zur Akzeptanz einer chronischen Behandlung: ACCEPT©, MAPI 2008 - Alle Rechte vorbehalten

Nebenwirkungen Ihres Medikaments

15. Hat Ihr Medikament bei Ihnen Nebenwirkungen?

Ja, und es fällt mir nicht leicht, dies zu akzeptieren.	Ja, aber es fällt mir leicht, dies zu akzeptieren.	Nein
☐1	☐2	☐3

16. Sind diese Nebenwirkungen unangenehm?

Ja, und es fällt mir nicht leicht, dies zu akzeptieren.	Ja, aber es fällt mir leicht, dies zu akzeptieren.	Nein	Ich habe keinerlei Nebenwirkungen.
☐1	☐2	☐3	☐4

17. Sind Sie durch diese Nebenwirkungen beeinträchtigt?

Ja, und es fällt mir nicht leicht, dies zu akzeptieren.	Ja, aber es fällt mir leicht, dies zu akzeptieren.	Nein	Ich habe keinerlei Nebenwirkungen.
☐1	☐2	☐3	☐4

18. Müssen Sie gegen die Nebenwirkungen Ihres Medikaments zusätzliche Medikamente einnehmen?

Ja, und es fällt mir nicht leicht, dies zu akzeptieren.	Ja, aber es fällt mir leicht, dies zu akzeptieren.	Nein	Ich habe keinerlei Nebenwirkungen.
☐1	☐2	☐3	☐4

19. Besteht bei Ihrem Medikament die Gefahr schwerer Nebenwirkungen für Ihre Gesundheit?

Ja, und es fällt mir nicht leicht, dies zu akzeptieren.	Ja, aber es fällt mir leicht, dies zu akzeptieren.	Nein	Ich weiss nicht.
☐1	☐2	☐3	☐4

Fragebogen zur Akzeptanz einer chronischen Behandlung: ACCEPT©, MAPI 2008 - Alle Rechte vorbehalten

Wirksamkeit Ihres Medikaments

20. Finden Sie, dass Ihr Medikament bei Ihnen wirkt?

Nein, und es fällt mir nicht leicht, dies zu akzeptieren.	Nein, aber es fällt mir leicht, dies zu akzeptieren.	Ja	Ich weiss nicht.
☐1	☐2	☐3	☐4

21. Finden Sie, dass Ihr Medikament Sie ausreichend schützt?

Nein, und es fällt mir nicht leicht, dies zu akzeptieren.	Nein, aber es fällt mir leicht, dies zu akzeptieren.	Ja	Ich weiss nicht.	Mein Medikament soll mich nicht schützen.
☐1	☐2	☐3	☐4	☐5

22. Hat Ihr Medikament eine schnelle Wirkung auf Ihre Krankheit?

Nein, und es fällt mir nicht leicht, dies zu akzeptieren.	Nein, aber es fällt mir leicht, dies zu akzeptieren.	Ja
☐1	☐2	☐3

Fragebogen zur Akzeptanz einer chronischen Behandlung: ACCEPT©, MAPI 2008 - Alle Rechte vorbehalten

ACCEPT - Switzerland/German - Version of 09 Oct 2018 - Mapi.
ID0295-TR-70971 / ACCEPT_AU3.0_deu-CH.doc

Ihr Medikament im Allgemeinen

23. Stimmen Sie der folgenden Aussage zu? „Mein Medikament hat mehr Vorteile als Nachteile."

Stimme überhaupt nicht zu	Stimme eher nicht zu	Stimme etwas zu	Stimme voll und ganz zu	Ich weiss nicht.
☐1	☐2	☐3	☐4	☐5

24. Wenn Sie die Vorteile und Nachteile Ihres Medikaments betrachten, sehen Sie es dann als akzeptable Lösung an?

Überhaupt nicht akzeptabel	Nicht sehr akzeptabel	Einigermassen akzeptabel	Voll und ganz akzeptabel	Ich weiss nicht.
☐1	☐2	☐3	☐4	☐5

25. Sind Sie überzeugt, dass es sich auf lange Sicht lohnt, ihr Medikament einzunehmen?

Überhaupt nicht überzeugt	Nicht wirklich überzeugt	Einigermassen überzeugt	Voll und ganz überzeugt	Ich weiss nicht.
☐1	☐2	☐3	☐4	☐5

Bitte überprüfen Sie, ob Sie alle Fragen beantwortet haben.

Herzlichen Dank für Ihre Mitarbeit.

Fragebogen zur Akzeptanz einer chronischen Behandlung: ACCEPT©, MAPI 2008 - Alle Rechte vorbehalten

ACCEPT - Switzerland/German - Version of 09 Oct 2018 - Mapi.
ID0295-TR-70971 / ACCEPT_AU3.0_deu-CH.doc
