# Supplementary material for: Micronutrient therapy for pyrroluria: a retrospective analysis of patient acceptance
Source: Arch Gynecol Obstet. 2026 Jan 6;313(1):14. doi: 10.1007/s00404-025-08252-8 (PMC12774977; doi:10.1007/s00404-025-08252-8)
Supplement: Supplementary file 2 — (PDF 174 KB) [file 404_2025_8252_MOESM2_ESM.pdf]

Fragebogen Kryptopyrrolurie (KPU)

Sehr geehrte Studienteilnehmerin,
Sie haben Sich bereit erklärt, an der Studie mit dem Titel «Akzeptanz und Wirksamkeit der Therapie bei Kryptopyrrolurie» teilzunehmen. Damit leisten Sie einen wichtigen Beitrag zur klinischen Forschung - besten Dank! Der folgende Fragebogen enthält 43 Fragen, deren Beantwortung etwa 15-20 Minuten beanspruchen.

Im folgenden Fragebogen werden Sie einige Male auf den Begriff „Mikronährstoff" stoßen. Damit Sie ein klares Verständnis davon haben, was man unter Mikronährstoffen versteht, sind hier die Substanzklassen mit den wichtigsten Beispielen aufgelistet:

Substanzklasse	Beispiele
Wasserlösliche Vitamine	Vit. B1 (Thiamin), B2 (Riboflavin), B3 (Nikotinamid), B5 (Pantothensäure), B6 (Pyridoxin), B12 (Cobalamin), Biotin, Vit. C, Folsäure
Fettlösliche Vitamine	Vit. A, E, D, K
Vitaminoide	Coenzym Q10, α-Liponsäure, β-Carotin
Mineralstoffe	Kalzium, Magnesium, Kalium, Natrium, Phosphor, Schwefel
Spurenelemente	Selen, Zink, Jod, Eisen, Kupfer, Chrom, Fluor
Aminosäuren	Tryptophan, Lysin, Arginin, Carnitin, Glutathion
Fettsäuren	Mehrfach ungesättigte Omega-3-Fettsäuren / Omega-6-Fettsäuren
Enzyme	Antioxidantien (z.B. Superoxid-Dismutase), Proteasen (z.B. Bromelain)
Sekundäre Pflanzenstoffe	Carotinoide, Phytohormone
Probiotika	Laktobazillen
Präbiotika	Unverdauliche Kohlenhydrate (Inulin, Oligofructose)
Ballaststoffe	Cellulose, Pektin, Lignin

1. Teilnahmebedingungen: Sind Sie…
    a. Volljährig

<u>Indikation</u>

2. Welche Beschwerden hatten Sie vor der Diagnose der Kryptopyrrolurie (KPU)? (Mehrfachauswahl möglich)
    a. Müdigkeit/Erschöpfung
    b. Ein- und/oder Durchschlafprobleme
    c. Prämenstruelles Syndrom (PMS)
    d. Unregelmässiger Zyklus
    e. Menstruationsbeschwerden
    f. Haarausfall
    g. Empfindlichkeit auf Sonnenlicht
    h. Schilddrüsenstörung
    i. Nahrungsmittelunverträglichkeit
    j. Migräne
    k. Reizdarm-/Reizmagen-Syndrom
    l. Aufmerksamkeitsdefizit-/Hyperaktivitätsstörung
    m. Arthrose
    n. Osteoporose
    o. Chronische Schmerzen
    p. Konzentrationsschwierigkeiten
    q. Stimmungsschwankungen

r. Medikamentenunverträglichkeit
s. Allergien
t. Autoimmunerkrankung
u. Überforderung im Alltag
v. Psychiatrische Erkrankung (Depression, Schizophrenie, Angststörung)
w. Sonstige (Textfeld)

(gar nicht – sehr stark ausgeprägt für jedes Symptom 1-4)

3. Haben Sie für die oben genannte(n) Beschwerde(n) bereits andere Therapien oder Medikamente ausprobiert?
    a. Ja
    b. Nein

Wenn Ja:

4. Wieviele? (Textfeld)

5. Über welchen Zeitraum haben Sie diese Therapie(n) eingenommen/durchgeführt?
    a. < 1 Monat
    b. 1-6 Monate
    c. 6-12 Monate
    d. 1-2 Jahre
    e. > 2 Jahre

6. War(en) diese Therapie(n) für Sie wirksam?
    a. Ja
    b. Nein

<u>Wissensstand</u>

7. Wo haben Sie das erste Mal von der Kryptopyrrolurie (KPU) gehört?
    a. Hausärztin/Hausarzt
    b. Frauenärztin/Frauenarzt
    c. Therapeut/in
    d. Internet
    e. Bekanntenkreis/Familie
    f. Noch nie davon gehört
    g. Sonstige (Textfeld)

8. Waren die Informationen, die Sie zu Ihrer Diagnose (Kryptopyrrolurie) erhalten haben, verständlich?
    a. Die Informationen waren sehr gut verständlich
    b. Die Informationen waren genügend verständlich
    c. Die Informationen waren nicht genügend verständlich
    d. Die Informationen waren gar nicht verständlich

9. Fühlen Sie sich gut informiert über das Krankheitsbild Kryptopyrrolurie (KPU)?
    a. Ich fühle mich sehr gut informiert
    b. Ich fühle mich genügend informiert
    c. Ich fühle mich nicht genügend informiert
    d. Ich fühle mich gar nicht informiert

Compliance

10. Haben Sie die KPU-Mikronährstoffe im Durchschnitt an mindestens 6 Tagen pro Woche eingenommen?
    a. Ja
    b. Nein

Akzeptanz (ACCEPT©)

11. Finden Sie, dass die Zubereitung Ihres Medikaments umständlich ist?
    a. Ja, und es fällt mir nicht leicht, dies zu akzeptieren
    b. Ja, aber es fällt mir leicht, dies zu akzeptieren
    c. Nein
    d. Mein Medikament erfordert keine Zubereitung

12. Finden Sie, dass die Medikamenteneinnahme umständlich ist?
    (Beispiele für die Medikamenteneinnahme: Schlucken, Injektion mittels Spritze, Inhalation durch die Nase usw.)
    a. Ja, und es fällt mir nicht leicht, dies zu akzeptieren
    b. Ja, aber es fällt mir leicht, dies zu akzeptieren
    c. Nein

13. Empfinden Sie die Form Ihres Medikaments als unangenehm?
    (Beispiele für Medikamentenformen: Tablette, Kapsel, Pulver in Beuteln, Spritze, Tropfen, Inhalator usw.)
    a. Ja, und es fällt mir nicht leicht, dies zu akzeptieren
    b. Ja, aber es fällt mir leicht, dies zu akzeptieren
    c. Nein

14. Nehmen Sie Ihr Medikament bereits seit längerer Zeit ein?
    a. Ja, und es fällt mir nicht leicht, dies zu akzeptieren
    b. Ja, aber es fällt mir leicht, dies zu akzeptieren
    c. Nein

15. Werden Sie Ihr Medikament für längere Zeit einnehmen müssen?
    a. Ja, und es fällt mir nicht leicht, dies zu akzeptieren
    b. Ja, aber es fällt mir leicht, dies zu akzeptieren
    c. Nein

16. Fühlen Sie sich dadurch eingeschränkt, dass Sie sich an die Einnahmen Ihres Medikaments erinnern müssen?
    a. Ja, und es fällt mir nicht leicht, dies zu akzeptieren
    b. Ja, aber es fällt mir leicht, dies zu akzeptieren
    c. Nein

17. Fühlen Sie sich dadurch eingeschränkt, dass Sie sich die Zeit nehmen müssen, Ihr Medikament aus der Apotheke zu holen?
    a. Ja, und es fällt mir nicht leicht, dies zu akzeptieren
    b. Ja, aber es fällt mir leicht, dies zu akzeptieren
    c. Nein

18. Fühlen Sie sich dadurch eingeschränkt, dass Sie sich daran erinnern müssen, Ihr Medikament mit sich zu nehmen?
    a. Ja, und es fällt mir nicht leicht, dies zu akzeptieren
    b. Ja, aber es fällt mir leicht, dies zu akzeptieren
    c. Nein

19. Fühlen Sie sich dadurch eingeschränkt, dass Sie Ihr Medikament immer bei sich haben?
    a. Ja, und es fällt mir nicht leicht, dies zu akzeptieren
    b. Ja, aber es fällt mir leicht, dies zu akzeptieren
    c. Nein
    d. Ich muss mein Medikament nie mitnehmen.

20. Muss Ihr Medikament auf Reisen unter speziellen Bedingungen aufbewahrt werden?
    a. Ja, und es fällt mir nicht leicht, dies zu akzeptieren
    b. Ja, aber es fällt mir leicht, dies zu akzeptieren
    c. Nein

21. Finden Sie, dass Sie viele Medikamente einnehmen müssen?
    a. Ja, und es fällt mir nicht leicht, dies zu akzeptieren
    b. Ja, aber es fällt mir leicht, dies zu akzeptieren
    c. Nein

22. Können Sie Ihr Medikament diskret einnehmen?
    a. Nein, und es fällt mir nicht leicht, dies zu akzeptieren.
    b. Nein, aber es fällt mir leicht, dies zu akzeptieren.
    c. Ja

23. Finden Sie, dass die regelmässige Einnahme Ihres Medikaments Teil Ihres normalen Alltags geworden ist?
    a. Nein, und es fällt mir nicht leicht, dies zu akzeptieren.
    b. Nein, aber es fällt mir leicht, dies zu akzeptieren.
    c. Ja
    d. Ich muss mein Medikament nicht regelmässig nehmen.

24. Fühlen Sie sich dadurch eingeschränkt, wie oft Sie Ihr Medikament nehmen müssen?
    a. Ja, und es fällt mir nicht leicht, dies zu akzeptieren
    b. Ja, aber es fällt mir leicht, dies zu akzeptieren
    c. Nein

Sicherheit (ACCEPT©)

25. Hat Ihr Medikament bei Ihnen Nebenwirkungen?
    a. Ja, und es fällt mir nicht leicht, dies zu akzeptieren
    b. Ja, aber es fällt mir leicht, dies zu akzeptieren
    c. Nein

26. Sind diese Nebenwirkungen unangenehm?
    a. Ja, und es fällt mir nicht leicht, dies zu akzeptieren
    b. Ja, aber es fällt mir leicht, dies zu akzeptieren

c. Nein
d. Ich habe keinerlei Nebenwirkungen.

27. Sind Sie durch diese Nebenwirkungen beeinträchtigt?
    a. Ja, und es fällt mir nicht leicht, dies zu akzeptieren
    b. Ja, aber es fällt mir leicht, dies zu akzeptieren
    c. Nein
    d. Ich habe keinerlei Nebenwirkungen.

28. Müssen Sie gegen die Nebenwirkungen Ihres Medikaments zusätzliche Medikamente einnehmen?
    a. Ja, und es fällt mir nicht leicht, dies zu akzeptieren
    b. Ja, aber es fällt mir leicht, dies zu akzeptieren
    c. Nein
    d. Ich habe keinerlei Nebenwirkungen.

29. Besteht bei Ihrem Medikament die Gefahr schwerer Nebenwirkungen für Ihre Gesundheit?
    a. Ja, und es fällt mir nicht leicht, dies zu akzeptieren
    b. Ja, aber es fällt mir leicht, dies zu akzeptieren
    c. Nein
    d. Ich weiss nicht.

<u>Wirksamkeit (ACCEPT©)</u>

30. Finden Sie, dass Ihr Medikament bei Ihnen wirkt?
    a. Nein, und es fällt mir nicht leicht, dies zu akzeptieren.
    b. Nein, aber es fällt mir leicht, dies zu akzeptieren.
    c. Ja
    d. Ich weiss nicht.

31. Finden Sie, dass Ihr Medikament Sie ausreichend schützt?
    a. Nein, und es fällt mir nicht leicht, dies zu akzeptieren.
    b. Nein, aber es fällt mir leicht, dies zu akzeptieren.
    c. Ja
    d. Ich weiss nicht.
    e. Mein Medikament soll mich nicht schützen.

32. Hat Ihr Medikament eine schnelle Wirkung auf Ihre Krankheit?
    a. Nein, und es fällt mir nicht leicht, dies zu akzeptieren.
    b. Nein, aber es fällt mir leicht, dies zu akzeptieren.
    c. Ja

<u>Bilanz (ACCEPT©)</u>

33. Stimmen Sie der folgenden Aussage zu? „Mein Medikament hat mehr Vorteile als Nachteile.“
    a. Stimme überhaupt nicht zu
    b. Stimme eher nicht zu
    c. Stimme etwas zu
    d. Stimme voll und ganz zu
    e. Ich weiss nicht.

34. Wenn Sie die Vorteile und Nachteile Ihres Medikaments betrachten, sehen Sie es dann als akzeptable Lösung an?
    a. Überhaupt nicht akzeptabel
    b. Nicht sehr akzeptabel
    c. Einigermassen akzeptabel
    d. Voll und ganz akzeptabel
    e. Ich weiss nicht.

35. Sind Sie überzeugt, dass es sich <u>auf lange Sicht</u> lohnt, ihr Medikament einzunehmen?
    a. Überhaupt nicht überzeugt
    b. Nicht wirklich überzeugt
    c. Einigermassen überzeugt
    d. Voll und ganz überzeugt
    e. Ich weiss nicht.

<u>Bilanz</u>

36. Haben Sie aktuell noch Beschwerden?
    a. Ja
    b. Nein

Wenn Ja:

37. Welche Beschwerden haben Sie aktuell? (Mehrfachauswahl möglich)
    a. Müdigkeit/Erschöpfung
    b. Ein- und/oder Durchschlafprobleme
    c. Prämenstruelles Syndrom (PMS)
    d. Unregelmässiger Zyklus
    e. Menstruationsbeschwerden
    f. Haarausfall
    g. Empfindlichkeit auf Sonnenlicht
    h. Schilddrüsenstörung
    i. Nahrungsmittelunverträglichkeit
    j. Migräne
    k. Reizdarm-/Reizmagen-Syndrom
    l. ADHS/ADS
    m. Arthrose
    n. Osteoporose
    o. Chronische Schmerzen
    p. Konzentrationsschwierigkeiten
    q. Stimmungsschwankungen
    r. Medikamentenunverträglichkeit
    s. Allergien
    t. Autoimmunerkrankung
    u. Überforderung im Alltag
    v. Psychiatrische Erkrankung (Depression, Schizophrenie, Angststörung)
    w. Sonstige (Textfeld)

(Gar nicht – sehr stark ausgeprägt für jedes Symptom 1-4)

38. Würden Sie die KPU-Mikronährstofftherapie weiterempfehlen?
    a. Ja
    b. Eher Ja
    c. Eher Nein
    d. Nein

39. Sind Sie an folgender weiterführender Diagnostik interessiert? (Mehrfachauswahl möglich)
    a. Mikronährstoffstatus
    b. Neurostressprofil
    c. Schilddrüsenprofil
    d. Toxische Metalle
    e. Nahrungsmittelunverträglichkeit
    f. Histaminose
    g. Störungen des Gastrointestinaltraktes
    h. Hormonstatus
    i. Mitochondrienparameter
    j. Nitrosativer Stress

<u>Allgemein</u>

40. Was ist Ihr höchster Bildungsabschluss?
    a. Kein Schulabschluss
    b. Grund-/Hauptschulabschluss
    c. Gymnasium / Fachmittelschule (FMS) / Berufsmittelschule (BMS)
    d. Abgeschlossene Berufslehre
    e. Bachelor
    f. Master
    g. Promotion / Habilitation

41. Welche der folgenden Kategorien beschreibt Ihren Erwerbsstatus am besten?
    a. 1-41 Stunden pro Woche (Teilzeit)
    b. 42 Stunden pro Woche und mehr (Vollzeit)
    c. Nicht erwerbstätig und auf der Suche nach Arbeit
    d. Nicht erwerbstätig und nicht auf der Suche nach Arbeit
    e. Altersrentnerin (AHV-Bezügerin)
    f. Nicht arbeitsfähig (IV-Bezügerin)

42. Wie hoch ist Ihr monatliches Einkommen (Netto)?
    a. < 5'000 CHF
    b. 5'000 – 10'000 CHF
    c. > 10'000 CHF
    d. Ich habe kein eigenes Einkommen

43. Wie ist Ihr aktueller Zivilstand?
    a. Ledig
    b. In eingetragener Partnerschaft
    c. Verheiratet
    d. Geschieden
    e. Verwitwet
