# Supplementary material for: Micronutrient therapy for pyrroluria: a retrospective analysis of patient acceptance
Source: Arch Gynecol Obstet. 2026 Jan 6;313(1):14. doi: 10.1007/s00404-025-08252-8 (PMC12774977; doi:10.1007/s00404-025-08252-8)
Supplement: Supplementary file 3 — (PDF 1653 KB) [file 404_2025_8252_MOESM3_ESM.pdf]

Kanton Bern
Canton de Berne

Gesundheits-, Sozial- und Integrationsdirektion
Kantonale Ethikkommission für die Forschung

Murtenstrasse 31
3010 Bern
Bern
+41 31 633 70 70 (Telefon)
+41 31 633 70 71 (Telefax)
info.kek.kapa@be.ch
www.be.ch/gsi

Dorothy Pfiffner
+41 31 633 70 77
dorothy.pfiffner@be.ch

GSI-KEK, Murtenstrasse 31, 3010 Bern

Frau
Prof. Dr. med. Petra Stute
Universitätsklinik für Frauenheilkunde
Inselspital Bern
Friedbühlstrasse 19
3010 Bern

Bern, 09.09.2021, NR

Verfügung der KEK Bern, Erstentscheid

**Project-ID**	2021-01026
**Projekttitel**	Akzeptanz der Therapie bei Kryptopyrrolurie (KPU)
**Master-/Doktorarbeit von**	Zingg, Lena Maria
**Projektleitung**	Prof. Dr. med. Petra Stute
**Sponsor**	Prof. Dr. med. Petra Stute
**Zentren**	• Prof. Dr. med. Petra Stute, Universitätsklinik für Frauenheilkunde, Bern

Entscheid

- ☒ Die Bewilligung wird erteilt
- ☐ Die Bewilligung wird mit Auflagen erteilt
- ☐ Die Bewilligung kann noch nicht erteilt werden
- ☐ Die Bewilligung wird nicht erteilt
- ☐ Auf das Gesuch wird nicht eingetreten

Klassifizierung

- ☒ Forschungsprojekt gemäss HFV, Kategorie: A
  - ☒ Forschung mit Personen
  - ☐ Weiterverwendung des biologischen Materials oder der gesundheitsbezogenen Personendaten
  - ☐ mit Verstorbenen
  - ☐ mit Embryonen / Föten
  - ☐ mit ionisierender Strahlung

Entscheidverfahren

☐ ordentliches Verfahren ☐ vereinfachtes Verfahren ☒ Präsidialverfahren

Die Ethikkommission bestätigt, dass sie nach ICH-GCP arbeitet.

Gebühren

Betrag: CHF 0.-- **Tarifcode:**

Gemäss der geltenden Gebührenordnung von swissethics. Rechnungsstellung folgt durch die Kantonale Gesundheits-, Sozial- und Integrationsdirektion (GSI).

Rechtsmittelbelehrung

Gegen diese Verfügung kann innert 30 Tagen seit Eröffnung bei der Gesundheits-, Sozial-und Integrationsdirektion des Kantons Bern Beschwerde erhoben werden. Die Beschwerdefrist kann nicht verlängert werden. Die Beschwerdeschrift ist im Doppel bei der Gesundheits-, Sozial-und Integrationsdirektion, Rathausgasse 1, Postfach, 3000 Bern 8 einzureichen.

Eine allfällige Beschwerde, die in mindestens zwei Exemplaren einzureichen ist, muss einen Antrag, die Angabe von Tatsachen und Beweismitteln, eine Begründung sowie eine Unterschrift enthalten; der angefochtene Entscheid und andere greifbare Beweismittel sind beizulegen.

Sie muss

(a) angeben, welche Entscheidung anstelle der angefochtenen Verfügung beantragt wird und
(b) aus welchen Gründen diese andere Entscheidung verlangt wird sowie
(c) die Unterschrift der beschwerdeführenden Partei oder der sie vertretenden Person enthalten.

Der Beschwerdeschrift beizulegen sind die Beweismittel, soweit sie greifbar sind, und die angefochtene Verfügung. (Art. 32 und 60 ff. des Gesetzes vom 23. Mai 1989 über die Verwaltungsrechtspflege [VRPG; BSG 155.21]).

Kopie an

☐ BAG
☒ DLF
☐ Andere

Prof. Dr. med. Christian Seiler
Präsident KEK Bern

Dr. sc. nat. Dorothy Pfiffner
Vizepräsidentin
Leiterin Wissenschaftliches Sekretariat

Anhang: -Pflichten des Sponsors/der Prüfperson oder der Projektleitung
-Mögliche Entscheide und ihre Bedeutung
-Eingereichte Dokumente, Stand vom 01.09.2021

Anhang

Pflichten des Sponsors/der Projektleitung

Einreichung Dokumente: revidierte Dokumente und neue Dokumente zur Studie/zum Projekt sollen ausschliesslich über das Web-Portal BASEC eingereicht werden, auf der entsprechenden Formularseite des betreffenden Gesuches. Obsolete Dokumente sind dabei zu entfernen und Datums- und Versionsangaben entsprechend zu ergänzen. Die erfolgten Änderungen müssen im Korrekturmodus abgefasst werden und zusätzlich als ‚clean'-Version eingereicht werden. Die Studieninformationen und -einwilligungen, das Protokoll und die Amendments müssen in durchsuchbaren PDF-Dateien eingereicht werden, insbesondere müssen gescannte Dokumente eine Texterkennung durchlaufen haben (OCR). Das unterschriebene und datierte Begleitschreiben muss die Antworten auf eventuell von der EK gestellte Fragen enthalten. Revidierte Dokumente sind auch den weiteren Zulassungsbehörden zuzustellen, sofern diese involviert sind.

Anmerkung: Die zuständige Ethikkommission überprüft im Rahmen des Bewilligungsverfahrens Aufklärungsbogen und Einwilligungserklärung in einer der Amtssprachen Deutsch, Französisch oder Italienisch. Aufklärungsbogen und Einwilligungserklärung in einer anderen Sprache werden von der Ethikkommission lediglich zur Kenntnis genommen. Für die korrekte Übersetzung ist der Sponsor oder die Projektleitung verantwortlich.

Meldepflichten: Die rechtlich bindenden Melde- resp. Bewilligungspflichten an die Ethikkommission für wesentliche Änderungen, einen vorzeitigen Studienabbruch, unerwünschte Ereignisse u.a. sind einzuhalten (Verordnungen des Bundes). Der Abschlussbericht ist spätesten ein Jahr nach Studienende der Ethikkommission einzureichen.

Registrierungspflicht: Der Sponsor muss – falls es sich um einen klinischen Versuch handelt – diesen in einem WHO-Primärregister oder im Register der Nationalen Medizinbibliothek der USA (clinicaltrials.gov) erfassen und anschliessend diese Nummer im BASEC-Portal eingeben. Die Übertragung der erforderlichen Daten in das Swiss National Clinical Trials Portal (SNCTP) kann nach Bewilligung der Ethikkommission und Zustimmung des Gesuchstellers automatisch erfolgen. Die Informationen über den klinischen Versuch sind in beiden Registern öffentlich zugänglich. Zusätzlich veröffentlicht swissethics wenige Informationen wie Titel, Projekttyp oder Leit-Ethikkommission aller durch die kantonalen Ethikkommissionen bewilligten Gesuche auf swissethics.ch (ausser Phase-I-Studien).

Mögliche Entscheide und ihre Bedeutung

Die Bewilligung wird erteilt: Das Vorhaben kann gemäss bewilligtem Forschungsplan und im Rahmen der anwendbaren rechtlichen Bestimmungen durchgeführt werden. Weitere Bewilligungspflichten (Swissmedic/BAG) sind zu beachten

Die Bewilligung wird mit Auflagen erteilt: Das Vorhaben kann gemäss bewilligtem Forschungsplan gestartet werden und im Rahmen der anwendbaren rechtlichen Bestimmungen durchgeführt werden. Die Auflagen sind zu erfüllen und die Gesuchsunterlagen innert 30 Tagen entsprechend anzupassen. Die revidierten Dokumente werden nach Einreichung im Präsidialverfahren geprüft. Weitere Bewilligungspflichten (Swissmedic/BAG) sind zu beachten

Die Bewilligung kann noch nicht erteilt werden: Das Vorhaben kann noch nicht gestartet werden. Die nachfolgenden Bedingungen sind zu erfüllen bzw. die Fragen zu beantworten und die revidierten Dokumente erneut bei der Ethikkommission einzureichen. Die Ethikkommission überprüft die revidierten Dokumente und erteilt die Bewilligung, wenn die Bedingungen erfüllt bzw. die Fragen zufriedenstellend beantwortet sind.

Die Bewilligung wird nicht erteilt: Das Vorhaben kann in der vorliegenden Form nicht durchgeführt werden. Eine Neueinreichung ist möglich.

Auf das Gesuch wird nicht eingetreten: Begründung siehe vorne, z.B. nicht zuständig oder nicht bewilligungspflichtig.

Eingereichte Dokumente für das Hauptzentrum

**Prof. Dr. med. Petra Stute, Universitätsklinik für Frauenheilkunde, Bern**		
Dokument	Dok.Datum	Version
**1. Cover Letter**		
coverletter-kpu-signed.pdf	19/04/2021	
**3. Participant information sheet and informed consent (ICF)**		
studieninformation-kpu-mit-a-nderungen.docx	30/08/2021	2
**4. Study plan (protocol), signed and dated**		
ethikantrag-kpu-mit-a-nderungen.docx	30/08/2021	2
**6. Investigator's CV, dated**		
cv-p-stute-en-sig.docx	12/06/2020	
**11. Other documents handed over to study participants**		
fragebogen-kpu.docx	27/04/2021	1
**12. Details on nature and scope/value of compensation for participants**		
There is no compensation for the participation in this study		
**14. Information on secure handling of biological material and personal data, and in particular on the storage thereof**		
see doc/cat: **4**, page/ref: **11**		
**39. Miscellaneous / Varia**		
stellungnahme.docx	01/09/2021	1
ksl-platzantrag-definitive-anmeldung-an-lehrveranstaltung.pdf	30/08/2021	1
